# Use and efficacy of antifibrinolytic agents in patients undergoing growth-friendly surgery for neuromuscular scoliosis

**DOI:** 10.1007/s43390-025-01214-9

**Published:** 2025-10-27

**Authors:** Wei Wu, Jagjot Dosanjh, John Smith, Peter Strum, Paul Sponseller, Ishaan Swarup, Pediatric Spine Study Group

**Affiliations:** 1 School of Medicine, University of California, San Francisco, CA USA; 2 Department of Orthopaedic Surgery, University of California, San Francisco, CA USA; 3 Department of Orthopaedics, The Orthopaedic Partners, Park City, UT USA; 4 Department of Pediatric Orthopaedic Surgery, Cincinnati Children’s Hospital, Cincinnati, OH USA; 5 Department of Orthopedic Surgery, Johns Hopkins University School of Medicine, Baltimore, MD USA; 6 Valley Forge, Valley Forge, PA USA; 7 UCSF Benioff Children’s Hospital Oakland, 747 52nd Street, Oakland, CA 94609 USA

**Keywords:** Growth freindly instrumentation, Neuromusuclar scoliosis, Antifibrinolytic agents, Scoliosis

## Abstract

**Introduction:**

There is a paucity of data on the use, efficacy, and safety of antifibrinolytic agents (AF) in patients with neuromuscular scoliosis undergoing growth-friendly instrumentation. Previous studies have shown mixed results of AF agents in young patients with neuromuscular conditions, and other authors have expressed concerns regarding adverse effects in this medically fragile population. The purpose of this study was to investigate the rate of use of AF agents for growth-friendly surgery in patients with neuromuscular scoliosis, and assess its impact on blood loss and transfusion requirements.

**Methods:**

This is a retrospective cohort study of patients from a multicenter spine study group with neuromuscular scoliosis that underwent an index growth-friendly procedure. Patients with a history of venous thromboembolism and those undergoing revision surgery or lengthening surgery were excluded. Perioperative data were collected including patient demographics, type of instrumentation, use and type of AF agent, estimated blood loss (EBL), use and volume of cell saver, and intraoperative blood transfusion. Univariate statistics were used to determine differences.

**Results:**

This study included 335 patients with a mean age of 7 years (SD: 2.6). Of these patients, 176 patients were managed with VEPTR/TGR instrumentation and 159 patients were managed with MCGR instrumentation. AF agents were used in 36% of index cases. In cases with AF use, TXA was the most frequently used agent (TXA:68%, ACA:21%). The use of AF increased over the study period from less than 10% before 2010 to 75% in 2020 (R^2^ = 0.31). There was no statistical difference in EBL between patients who received AF agents compared to patients that did not receive AF agents (AF = 184.9 ml, no AF = 103 ml, p = 0.23). In addition, there was no difference in cell saver volume (AF = 127 ml, no AF = 145 ml, p = 0.88). The overall rate of intraoperative blood transfusion was low (8.5%). In this cohort, there was no significant difference in transfusion rates between groups (AF = 7.6%, no AF = 8.7%, *p* = 0.7). There was a high rate of postoperative blood transfusion (51.4%) in this cohort; however, there was no significant difference in postoperative transfusion rates between groups (AF = 62.1%, no AF = 50.5%, *p* = 0.62).

**Conclusion:**

AF agents are being used for patients undergoing growth-friendly procedures with TXA being the most commonly used AF. However, there is no significant difference in EBL, cell saver volume, and intraoperative or postoperative transfusion rates between patients that do or do not receive AF agents for these procedures. Additional studies are needed to validate these results, as well as determine their efficacy, safety, and value in this medically fragile group.

## Introduction

Neuromuscular scoliosis (NMS) is spinal deformity due to underlying neurologic or muscular abnormalities that causes a curvature of the spine. It is diagnosed with a 10-degree or greater lateral deviation of the spine in the coronal plane [1]. NMS can be caused as a result of multiple diagnoses, such as myelomeningocele, spinal muscular atrophy, spinal cord injury, and cerebral palsy [2]. NMS can occur at any age and progressive curves may limit an individual's mobility, ability to sit, and may impact cardiopulmonary function [2]. Current management of NMS includes observation, non-surgical intervention, and surgical interventions. The efficacy of non-surgical interventions, such as braces and custom seating systems, has proven to be limited. Most patients undergoing surgical treatment for NMS undergo posterior spinal instrumentation [2].

Vertical expandable prosthetic titanium rib (VEPTR) instrumentation, traditional growing rods (TGR), and magnetically controlled growing rods (MCGR) instrumentation are types of growth-friendly procedures in younger children with NMS. These measures are alternatives to spinal fusion and permit spine and lung development. VEPTR instrumentation couples to the pelvis or ribs vertically. It is distracted to expand the chest wall while managing the curvature of the spine [3]. TGR and VEPTR instrumentation allows for focal exertion of forces due to their direct coupling to the spine and thoracic cavity [3]. MCGR was developed to limit multiple surgeries, such as every 6–8 months in TGR distraction [4]. MCGR is an outpatient spinal distraction system that works using an external magnet field over an internally placed magnet to extend the rod [4]. This non-invasive technique aims to lower surgical complications that result from the more invasive TGR [4]. Older patients may undergo posterior spinal fusion (PSF).

Posterior spinal instrumentation in patients with NMS, however, has been associated with extensive blood loss and perioperative morbidity. The need for allogenic transfusion in NMS patients is 7.8 times greater than the need for transfusion in idiopathic scoliosis patients [5]. Transfusions add to healthcare costs and increase the risk of complications, such as pulmonary edema, surgical site infection, and coagulopathy [5]. Intraoperative use of antifibrinolytic (AF) agents has become increasingly popular to reduce surgical bleeding and limit the complications from transfusions.

The use of preoperative and intraoperative tranexamic acid (TXA) has shown to be significantly effective on both morbidity and mortality in various orthopaedic procedures. TXA produces secondary hemostasis by blocking lysine binding sites on plasminogen molecules which results in the stability of fibrin [6]. TXA is associated with a decreased need for transfusion and increased survival in bleeding patients [5]. Previous studies on aminocaproic acid (ACA) also demonstrate an association with a significant reduction in total blood loss, hemoglobin drop, and need for transfusion [7]. Similar to TXA, ACA exerts its hemostatic effect by preventing the breakdown of fibrin. ACA competitively binds to plasminogen which prevents plasminogen’s conversion to plasmin via fibrin bondage [8]. However, there are insufficient data on the use, efficacy, and safety of antifibrinolytic agents in patients with NMS undergoing growth-friendly instrumentation. Furthermore, previous research on young patients with neuromuscular disorders has also yielded conflicting outcomes when it comes to AF agents. In a study assessing the impact of intravenous (IV) ACA on blood loss and transfusion requirements after bilateral varus rotational osteotomy, there was no difference in calculated blood loss or transfusion rates between patients who received IV ACA versus placebo [9]. Therefore, high-quality studies with large sample sizes are needed to determine the efficacy and safety of AFs in patients with NMS undergoing growth-friendly instrumentation.

The purpose of this multi-centered, retrospective cohort study was to investigate the rate of use of AF agents for growth-friendly instrumentation in patients with NMS and assess its impact on blood loss and transfusion requirements. We hypothesized that antifibrinolytic agents are commonly used but may not be necessary in reducing both surgical blood loss and the need for transfusions in this patient population.

## Methods

We performed a retrospective study using data from a multicenter study group. IRB was obtained at individual institutions. We included consecutive patients with NMS between the ages of 0–18 that underwent index growth-friendly instrumentation from 1995 to 2020. Patients with a history of venous thromboembolism and those undergoing revision surgery were excluded.

Demographic data and perioperative measurements were collected; these include preoperative and postoperative hemoglobin and hematocrit, use of AF, estimated blood loss (EBL), use of cell saver, administration of blood products, and surgical levels. Patients were grouped into VEPTR/TGR or MCGR based on type of instrumentation. TXA, when administered, was most commonly dosed at 100 mg/kg loading at the start of the surgery, followed by a maintenance dose of 10 mg/kg/h until skin closure. However, dosing of TXA and transfusion criteria were not standardized across sites. EBL was determined by the primary surgeon and anesthesia and recorded in the database. The amount of suction drainage and cell saver return was also recorded.

Descriptive statistics were used to summarize the cohort. Statistical differences in EBL and cell saver volume between groups were analyzed using Student's t test. Rates of blood product transfusion were compared using Chi-square. All analyses were performed in Microsoft Excel (v. 16).

## Results

**Fig. 1:**
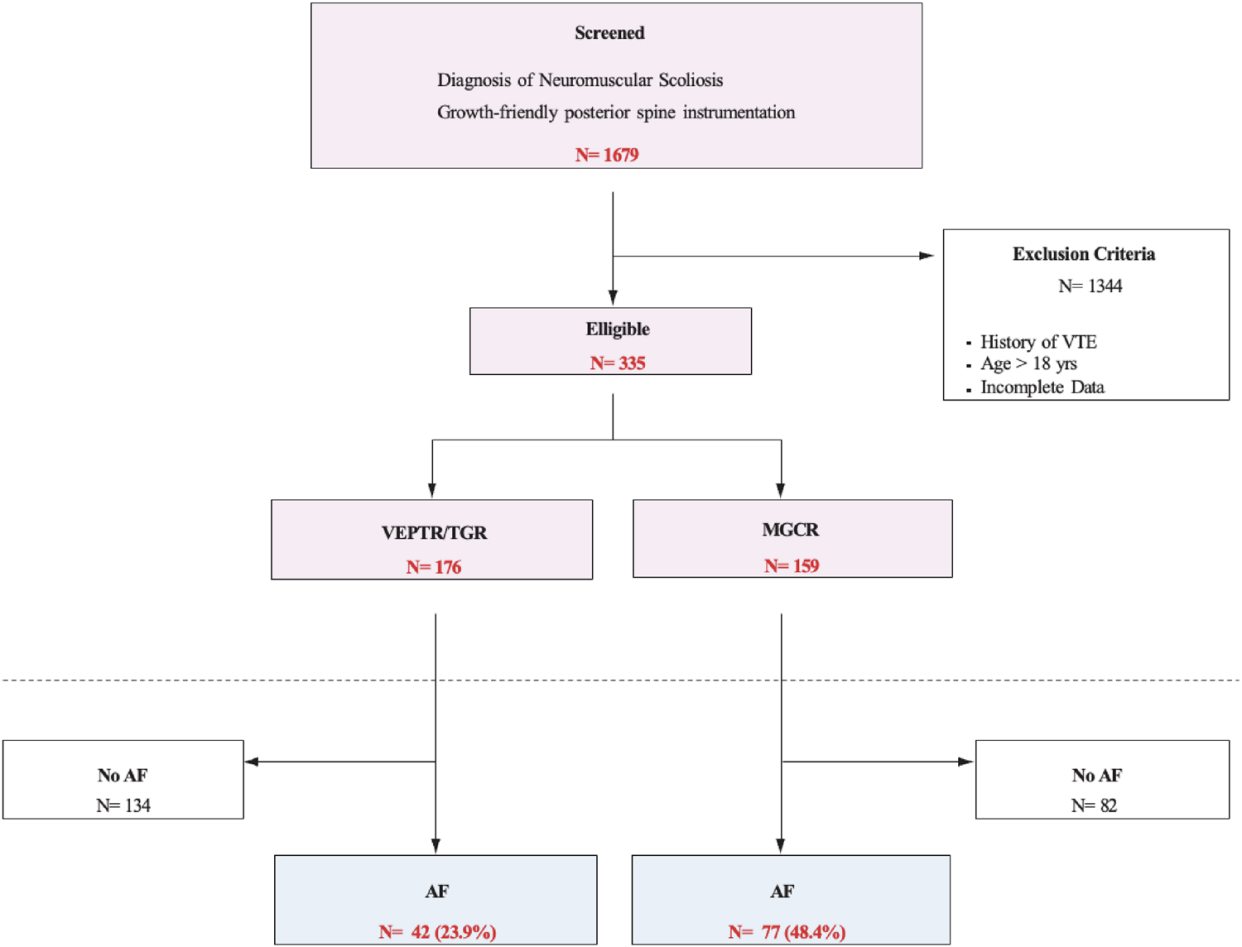
Flowchart of study population leading to final cohort

This study included 335 patients (Fig. 1). In total, 176 patients underwent VEPTR/TGR instrumentation and 159 patients underwent MCGR instrumentation (Table 1). The average age of patients was 7.2 years (SD = 2.62) and baseline demographic characteristics were compared between VEPTR/TGR versus MCGR groups (Table 1). Patients ranged in age from 1 to 17 years and subgroup comparisons based on median age (< 7.2 vs. ≥ 7.2 years) did not reveal any significant differences in estimated blood loss or transfusion rates. The most common underlying etiology was cerebral palsy (VEPTR/TGR: 17%, MCGR: 32.7%), and 48.1% of patients were non-ambulatory (VEPTR/TGR: 46.6%, MCGR: 49.7%). A full breakdown of underlying neuromuscular diagnoses showed that cerebral palsy was most common (77%), followed by muscular dystrophy (12%), spina bifida (6%), and other conditions (5%).

**Table 1:** Baseline characteristics of study cohort

	VEPTR/TGR	MCGR	Total	*P* value
Age (yrs)	7.1 (1–17)	7.2 (1–12)	7.2	*p* = 0.88
Female (%)	56.3	52.2	54.3	*p* = 0.45
Male (%)	43.7	47.8	45.7	*p* = 0.29
BMI (kg/m^2^)	16.7	16.4	16.6	*p* = 0.58
Cerebral palsy (%)	17.0	32.7	24.4	*p* = 0.0008
Preop non-ambulatory (%)	46.6	49.7	48.1	*p* = 0.57
Preop Hgb (g/dL)	12.8	20.2	16.5	*p* = 0.004

**Fig. 2:**
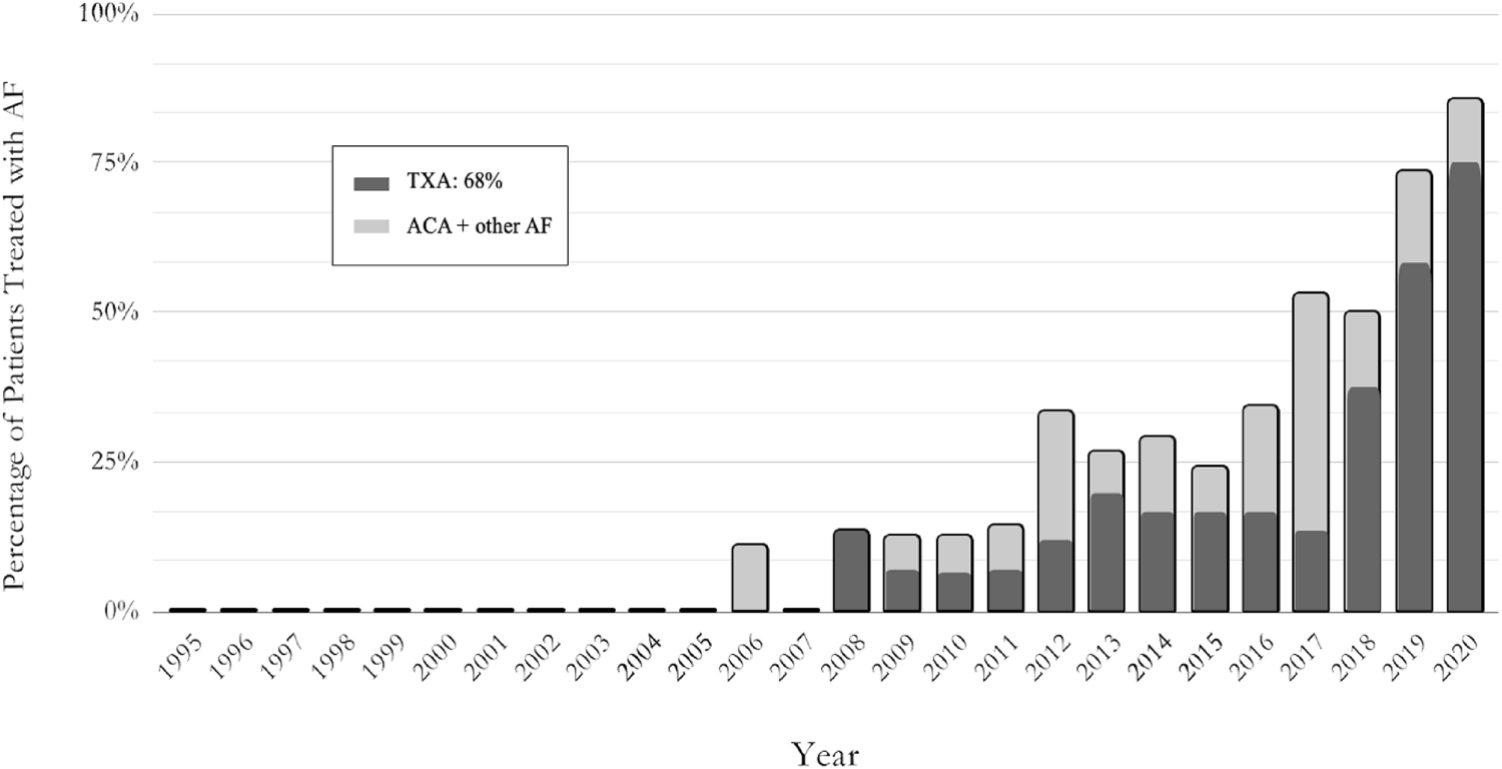
Trend in AF use between 1995 and 2020

**Fig. 3:**
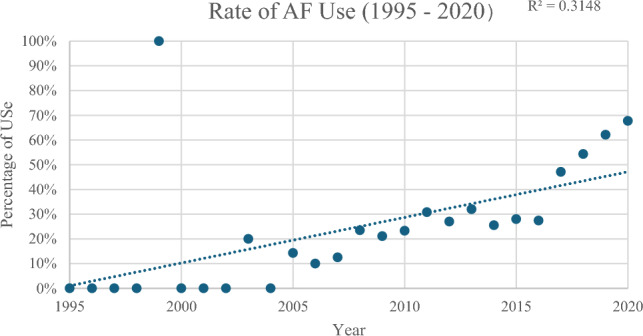
Trend analysis for rate of AF use between 1995 and 2020

Approximately 36% of patients received AFs; however, the percentage of patients treated with AF increased over the study period from less than 10% before 2010 to 75% in 2020 (Fig. 2). Trend analysis of AF use between 1995 and 2020 had an R^2^ value of 0.31 (Fig. 3). AF utilization rate was higher in patients with MCGR vs. VEPTR/TGR (48% vs. 24%). TXA was the most commonly type of AF agent used in this cohort (TXA:68%, ACA:21%). Estimated blood loss was similar between these groups (TXA: 153.2 mL vs. ACA: 164.7 mL, *p* = 0.78), and postoperative transfusion rates did not differ meaningfully.

### Intraoperative blood loss

The average EBL within the cohort was 164.3 ml. There was no statistical difference in EBL between patients who received AF compared to those who underwent surgery without AF (AF = 184.9 ml, no AF = 159.3 ml, *p* = 0.23; Table 2). The average cell saver volume within the cohort was 136.7 ml. Similar to EBL, there was no statistical difference in cell saver volume between patients who received AF compared to those that did not receive AF (AF = 127 ml, no AF = 145 ml, *p* = 0.88).

**Table 2:** Intraoperative blood loss—AF vs no AF

	N	EBL (ml)	Cell Server (ml)
No AF	216	159.3	145.0
AF	119	184.9	127.0
All Patients	335	164.3	136.7
		*p* = 0.229	*p* = 0.883

In analysis of estimated blood loss by instrumentation type and AF use, the mean EBL was 195.0 mL for MCGR and 162.6 mL for VEPTR/TGR (*p* = 0.32) in the AF group. In the non-AF group, instrumentation type was again not associated with significantly higher blood loss as EBL for MCGR compared to VEPTR/TGR was 161.3 mL and 158.0 mL, respectively (*p* = 0.91).

### Perioperative transfusion

The overall rate of intraoperative blood transfusion was low (8.5%). There was no statistically significant difference in transfusion rates between groups (AF = 7.6%, no AF = 8.7%, *p* = 0.7; Table 3). The use of AF was not associated with a significantly lower odds of transfusion (OR = 0.85, *p* = 0.35). There was a high rate of postoperative blood transfusion (51.4%) in this cohort; however, there was no significant difference in postoperative transfusion rates between groups (AF = 62.1%, no AF = 50.5%, *p* = 0.62). No adverse events such as seizures or thromboembolic complications were reported in patients who received antifibrinolytic agents.

**Table 3:** Transfusion rate—AF vs no AF

	N	Intraop Transfusion (%)	Postop Transfusion (%)
No AF	216	8.7	50.5
AF	119	7.6	62.1
All Patients	335	8.5	51.4
		*p* = 0.696	*p* = 0.623

## Discussion

AF agents are commonly used in pediatric spine surgery. The use and efficacy of these agents is relatively unknown in patients with neuromuscular scoliosis undergoing growth-friendly procedures. These procedures are typically performed on younger children and performed via smaller incisions with presumably decreased blood loss. Previous studies have suggested varying efficacy in younger patients with neuromuscular conditions and this study sought to investigate the use and efficacy of these agents in growth-friendly procedures. We found that the use of AF agents is increasing over time. They are used in the majority of growth-friendly cases, and TXA is the most commonly used AF agent. However, there is no significant difference in EBL, cell saver volume, or transfusion requirements during and after surgery.

The EBL in these cases is lower than PSF. This is likely related to decreased soft-tissue dissection and bony work in patients undergoing growth-friendly procedures. There are several previous studies that have shown limited efficacy of AF agents in patients with neuromuscular conditions undergoing orthopaedic procedures. For example, Majid et al. and Nazareth et al. showed no difference in blood loss or transfusion requirements in CP patients treated with TXA in the setting of hip reconstruction [10, 11]. Similarly, Swarup et al. showed in a prospective study that aminocaproic acid did not decrease calculated blood loss or transfusion requirements in a similar population [9]. Some reasons for this finding could relate to the protective coagulation response in younger, pediatric patients [12]. Additionally, growth-friendly procedures are less invasive than other procedures such as PSF or total joint arthroplasty, which have been more commonly studied and where AF agents have been shown to be efficacious. When analyzed by instrumentation type and AF use, MCGR and VEPTR/TGR procedures were associated with similar blood loss. This suggests that differences in instrumentation type alone may not impact blood loss in growth-friendly procedures, regardless of AF intervention. Instead, other factors, such as patient characteristics and institutional practices, may have a greater influence on intraoperative bleeding.

It is also important to note that there is cost and risk associated with AF use. Specifically, TXA has been associated with postoperative seizures in pediatric patients [13, 14], and several patients with neuromuscular conditions already have underlying seizure disorders. It is also contraindicated in patients with a history of thromboembolic events. In the absence of clear benefit, routine use of these agents may not be justified given increased cost and risk of potentially fatal side effects such as thromboembolism in this fragile population. Importantly, no adverse events consistent with seizures or thromboembolic complications were reported in our cohort following the use of antifibrinolytics.

This study has several limitations. First, we had a large sample size but lacked more specific clinical data. For example, we did not have consistent perioperative blood counts or anesthesia records to calculate blood loss. We relied on the medical record and surgeon/anesthesia estimates, which may be susceptible to bias and error. Additionally, we are unable to calculate estimated blood volume due to limited demographic data. Second, we excluded patients with incomplete medical records which may introduce selection bias. Finally, these procedures were performed at mostly tertiary medical centers that are part of the study group, and the results may not be generalizable to all settings. Institutional variability in transfusion thresholds, clinical decision-making, and perioperative protocols likely contributed to the observed differences in postoperative transfusion rates. Because this was a retrospective study across multiple centers, transfusion practices were not standardized. In addition, the relatively small sample sizes for non-cerebral palsy conditions limited our ability to perform condition-specific subgroup analyses.

In conclusion, AF agents are commonly used for growth-friendly procedures in patients with NMS. There is no difference in EBL, cell saver volume, or transfusion requirements in patients that did or did not receive AFs in patients undergoing this procedure. While no prospective or randomized studies are currently planned, future prospective studies with placebo control, randomization, and inclusion of AF-associated adverse events are needed to define the indications for AF use in this critical patient population.

## Data Availability

This data is not publically available.
